# Multiwalled Carbon Nanotube Reinforced Bio-Based Benzoxazine/Epoxy Composites with NIR-Laser Stimulated Shape Memory Effects

**DOI:** 10.3390/nano9060881

**Published:** 2019-06-14

**Authors:** Wassika Prasomsin, Tewarak Parnklang, Chaweewan Sapcharoenkun, Sunan Tiptipakorn, Sarawut Rimdusit

**Affiliations:** 1 Polymer Engineering Laboratory, Department of Chemical Engineering, Faculty of Engineering, Chulalongkorn University, 254 Phayathai Road, Pathumwan, Bangkok 10330, Thailand; w.prasomsin@gmail.com (W.P.); ParnklangT@gmail.com (T.P.); 2 National Nanotechnology Center (NANOTEC), National Science and Technology Development Agency (NSTDA), 111 Thailand Science Park, Phahonyothin Road, Khlong Nueng, Khlong Luang, Phatum Thani 12120, Thailand; chaweewan@nanotec.or.th; 3 Department of Chemistry, Faculty of Liberal Arts & Science, Kasetsart University, Nakhon Pathom 73140, Thailand; faassntk@ku.ac.th; 4 Research Network NANOTEC-CU on Advanced Structural and Functional Nanomaterials, Chulalongkorn University, Bangkok 10330, Thailand

**Keywords:** bio-based benzoxazine, multiwalled carbon nanotubes, shape memory polymers, NIR actuation, composites, renewable materials

## Abstract

Smart materials with light-actuated shape memory effects are developed from renewable resources in this work. Bio-based benzoxazine resin is prepared from vanillin, furfurylamine, and paraformaldehyde by utilizing the Mannich-like condensation. Vanillin-furfurylamine-containing benzoxazine resin (V-fa) is subsequently copolymerized with epoxidized castor oil (ECO). When the copolymer is reinforced with multiwalled carbon nanotubes (MWCNTs), the resulting composite exhibits shape memory effects. Molecular characteristics of V-fa resin, ECO, and V-fa/ECO copolymers are obtained from Fourier transform infrared (FT-IR) spectroscopy. Curing behavior of V-fa/ECO copolymers is investigated by differential scanning calorimetry. Dynamic mechanical properties of MWCNT reinforced V-fa/ECO composites are determined by dynamic mechanical analysis. Morphological details and distribution of MWCNTs within the copolymer matrix are characterized by transmission electron microscopy. Shape memory performances of MWCNT reinforced V-fa/ECO composites are studied by shape memory tests performed with a universal testing machine. After a significant deformation to a temporary shape, the composites can be recovered to the original shape by near-infrared (NIR) laser actuation. The shape recovery process can be stimulated at a specific site of the composite simply by focusing NIR laser to that site. The shape recovery time of the composites under NIR actuation is four times faster than the shape recovery process under conventional thermal activation. Furthermore, the composites possess good shape fixity and good shape recovery under NIR actuation.

## 1. Introduction

Petroleum and their products from refining processes are employed as energy resources and raw materials for industrial production of plastics, cosmetics, fuel, and rubber [1]. Considerably rapid rate of petroleum consumption have inevitably led to a shortage of petroleum supply, air pollution problems generated from petroleum refining, global warming, and other related environmental issues [2]. Therefore, renewable starting materials from bio-based resources are increasingly employed as alternatives to petroleum-based materials. Bio-based materials are attractive due to their biodegradability, renewability, and low ecotoxicity [3]. Recently, bio-based materials have been utilized for producing food packaging [4], implantable medical devices, bioactive substances, textiles, paints, pharmaceuticals, automotive, ink, nail care, and cosmetics [5].

Polybenzoxazines are a novel kind of thermosetting phenolic resin derived from ring-opening polymerization of their precursors [6]. Benzoxazine monomers could be prepared from limitless choices of phenolic compounds, primary amines, and formaldehyde through the Mannich-like condensation, due to their wide molecular design flexibility [7,8,9]. The monomers exhibit near-zero volumetric shrinkage or expansion upon polymerization. Cross-linked polybenzoxazines possess exceptionally high glass transition temperature (*T*_g_) and high thermal stability. Even though polybenzoxazines contain polar functional groups, these thermosetting polymers exhibit low water absorption due to complex hydrogen bond formation within their polymeric networks [9,10]. Furthermore, high performance materials could be obtained by alloying polybenzoxazines with various resins [11]. Benzoxazine resins could act as a latent curing agent for epoxy and benzoxazine/epoxy copolymers exhibiting synergism in *T*_g_ [12,13]. Moreover, the resins could also act as a permanent segment in benzoxazine/epoxy shape memory polymers [14]. Polybenzoxazines possess unique supramolecular structures and functionality that could be potentially employed for developing smart materials [15]. Polybenzoxazine-based smart materials including shape memory polymers, self-healable polymers, electrochemically activated coatings, and electrochromic resins were successfully fabricated [16].

Recently, benzoxazine resins have been successfully prepared from renewable materials including vanillin [17,18], eugenol [19], cardonol [20], furfurylamine [21,22], stearylamine [23], and guaiacol [22]. In this work, vanillin and furfurylamine were selected as starting materials for bio-based benzoxazine resin preparation. Vanillin-furfurylamine-containing benzoxazine (V-fa) showed a low onset of curing temperature (179 °C), very high *T*_g_ due to highly cross-linked structures of the cured polymer, and high char yield compared to petroleum based benzoxazines [17]. Vanillin could be synthesized from lignin and other renewable resources including eugenol, iso-eugenol, ferulic acid, and sugar [24]. Aldehyde groups in vanillin offered additional chemical functionality to vanillin-based benzoxazine monomers [18]. Furfural derived from byproducts of agriculture including corncobs and wheat bran were employed as the starting material for producing furfurylamine [3]. Furfurylamine possessed methylene elasticity and crosslinkable furan rings that could be employed for enhancing *T*_g_ and thermal stability of high performance benzoxazine thermosets through the formation of furfurylamine Mannich bridge networks during polymerization [25].

Shape memory polymers (SMPs) are smart materials possessing the ability to recover their original permanent shape after significant deformation by exposing to an appropriate stimulus [26], such as light induction [27,28,29,30,31], temperature change [32,33,34], magnetic field [35,36], or pH variation [37]. SMPs have been demonstrated in several potential applications including switches and sensors, shrinkable tubes, auto repairing and self-healing polymers, biomedical sensors and devices, smart textiles, and deployable structures for aircraft and spacecraft [38]. Furthermore, SMPs have been used to substitute shape memory metallic alloys (SMAs) because they possess distinctive advantages including excellent processing, light weight, low cost, easy control of activation temperature, and great flexibility [39]. Light-triggered shape memory effects have become increasingly attractive due to the spatially controlled and remote activation at low operating temperatures. In addition, light-triggered processes can be paused and resumed as needed by turning the light off or tuning the light on. Light-triggered SMPs should contain specific kinds of fillers or additives that are capable of converting optical energy to heat to generate the photothermal effect, for example organic dyes, gold nanoparticles (AuNPs), gold nanorods (AuNRs), and carbon nanotubes (CNTs) [40]. Recently, photothermal effects utilizing carbon nanotubes as a NIR light absorber have been increasingly employed as an external stimulus for triggering shape memory behavior. Yi et al. [41] developed NIR-triggered SMPs based on hyperbranched polyurethane composites containing 1D thin-walled carbon nanotubes and 2D reduced graphene oxide. They found that the optically active 1D thin-walled carbon nanotubes efficiently transferred the laser-induced thermal energy to the polymer matrix. Koerner et al. [42] demonstrated a very fast strain recovery within 5 seconds of MWCNT-Morthane nanocomposites with NIR light triggering. Anisotropy of carbon nanotubes might effectively enhance shape memory effects of polymer composites [40]. In addition, MWCNTs could effectively transform NIR radiation into thermal energy when compared to single-walled carbon nanotubes (SWCNTs), resulting in higher temperature enhancement at the same mass concentrations [43].

It is the purpose of this study that renewable resources are utilized as raw materials for light-stimulated shape memory composite fabrication. Bio-based benzoxazine resin is synthesized from the Mannich-like condensation of vanillin, furfurylamine, and paraformaldehyde. The bio-based benzoxazine resin is further copolymerized with epoxidized castor oil. To impart light-actuated shape memory effects, the resulting copolymer is filled with MWCNTs. MWCNTs play a role as NIR-laser absorber, transforming electromagnetic energy to thermal energy. Shape memory effects of MWCNT reinforced bio-based benzoxazine/epoxy composites can be remotely activated with NIR laser. Furthermore, the shape recovery process of the MWCNT reinforced composites can be selectively activated at specific sites, demonstrating the high level of controllability. The MWCNT reinforced composites show good shape fixity up to 93% and good shape recovery up to 98%.

## 2. Materials and Methods

### 2.1. Materials

Vanillin (99%) and furfurylamine (99%) were purchased from Sigma-Aldrich Pte. Ltd. (St. Louis, MO, USA). Paraformaldehyde (AR grade) was purchased from Merck Co., Ltd. (Darmstadt, Germany). Epoxidized castor oil (ECO) was supplied by Aditya Birla Chemicals Thailand Ltd. (Rayong, Thailand). MWCNTs with the outer diameter of 12.9 nm and the tube length of 3 to 12 μm were purchased from Nano Generation Company Limited. (Chiang Mai, Thailand). All chemicals were used as received.

### 2.2. Synthesis of Benzoxazine Monomer

Benzoxazine monomer (V-fa) was synthesized using vanillin, furfurylamine, and paraformaldehyde at a 1:1:2 molar ratio based on the solventless method [17]. The three reactants were mixed at 105 °C for 1 h. The V-fa resin was a transparent yellow viscous liquid at room temperature.

### 2.3. Preparation of V-fa/ECO Copolymer Reinforced with MWCNTs

The benzoxazine monomer (V-fa) was blended with ECO to provide V-fa/ECO copolymer. The mass concentration of ECO was fixed at 50 wt%. The mixture was preheated at 105 °C in an aluminum pan and thoroughly mixed until a homogeneous mixture was obtained. MWCNTs at 0, 0.1, 0.3, and 0.5 wt% might be gradually added to the homogeneous mixture while stirring to fabricate the MWCNT reinforced V-fa/ECO composite. The molten resin mixture dispersed with 0, 0.1, 0.3, and 0.5 wt% MWCNTs were poured into an aluminum mold and step-cured in an air-circulated oven at 150 °C for 1 h, 160 °C for 1 h, 170 °C for 2 h, and 180 °C for 2 h. The samples were finally left to cool down to room temperature and were ready for material characterizations.

### 2.4. Characterization Methods

#### 2.4.1. Differential Scanning Calorimetry

Curing behavior of the V-fa/ECO copolymer was obtained from differential scanning calorimeter (DSC, model DSC1 module from Mettler-Toledo (Thailand) Ltd. (Bangkok, Thailand)). Each sample with a mass in a range of 5 to 10 mg was sealed in an aluminum pan covered with a lid. The sample was dynamically scanned from 25 to 300 °C with a heating rate of 10 °C min^−1^ under a nitrogen flow rate of 50 mL min^−1^. The degree of conversion (%) of a sample was determined according to the relationship in Equation (1):

(1) $$\mathrm{conversion}\;(\%)=(1-\frac{H_{\mathrm{rxn}}}{Ho})\times 100$$

where *H*_rxn_ is the heat of reaction of the partially cured sample and *H*_o_ is the heat of reaction of the uncured resin mixture. The heat of reaction was determined from the area under the exothermic peak of DSC thermograms.

#### 2.4.2. FT-IR Spectroscopy

Molecular characteristics and network formation of all samples were studied by a Spectrum GX FT-IR spectrometer from Perkin Elmer with an ATR accessory (Waltham, MA, USA). All spectra were taken as a function of time with 64 scans at a resolution of 4 cm^−1^ and wavenumber ranging from 4000 to 650 cm^−1^. For viscous liquid V-fa/ECO mixtures, a small amount of mixture was casted as a thin film on a potassium bromide (KBr) window and its FT-IR spectrum was obtained.

#### 2.4.3. Dynamic Mechanical Analysis

Dynamic mechanical analyzer (DMA) model DMA242 from Netzsch, Inc. (Bavaria, Germany) was used to determine a storage modulus (*E*′) and loss tangent (tan δ) of the V-fa/ECO copolymer reinforced with various mass concentrations of MWCNTs in the range from 0 to 0.5 wt%. The sample in rectangular shape with dimensions of 5 mm × 50 mm × 2 mm was tested using a tension mode at the frequency of 1 Hz, a strain amplitude of 5 µm, and a heating rate of 2 °C/min from −100 °C to 200 °C under nitrogen atmosphere with a gas purging rate of 80 mL min^−1^. The gauge length was approximately 10 mm for all samples. *T*_g_ was taken as the peak maximum on the loss tangent curve in the DMA spectrum.

#### 2.4.4. Transmission Electron Microscopy

The morphology and dispersion of MWCNTs in the polymer matrix was investigated by a JEM-2100 transmission electron microscope (JEOL Ltd. (Tokyo, Japan)) operated at 200 kV. Pure MWCNTs were sonicated in water and a droplet of the suspension was put onto a 200-mesh carbon-coated copper grid for observation. Dimensions of MWCNTs were measured directly from transmission electron micrographs using ImageJ software (a Java program developed by the National Institute of Mental Health) [44]. An ultra-thin film of a composite specimen was prepared by an ultramicrotome Leica EM UC7 (Leica Microsystems (SEA) Pte. Ltd. (Singapore)) at room temperature with a section thickness of 100 nm and placed onto a copper grid.

#### 2.4.5. Shape Memory Performances

**Figure 1:**
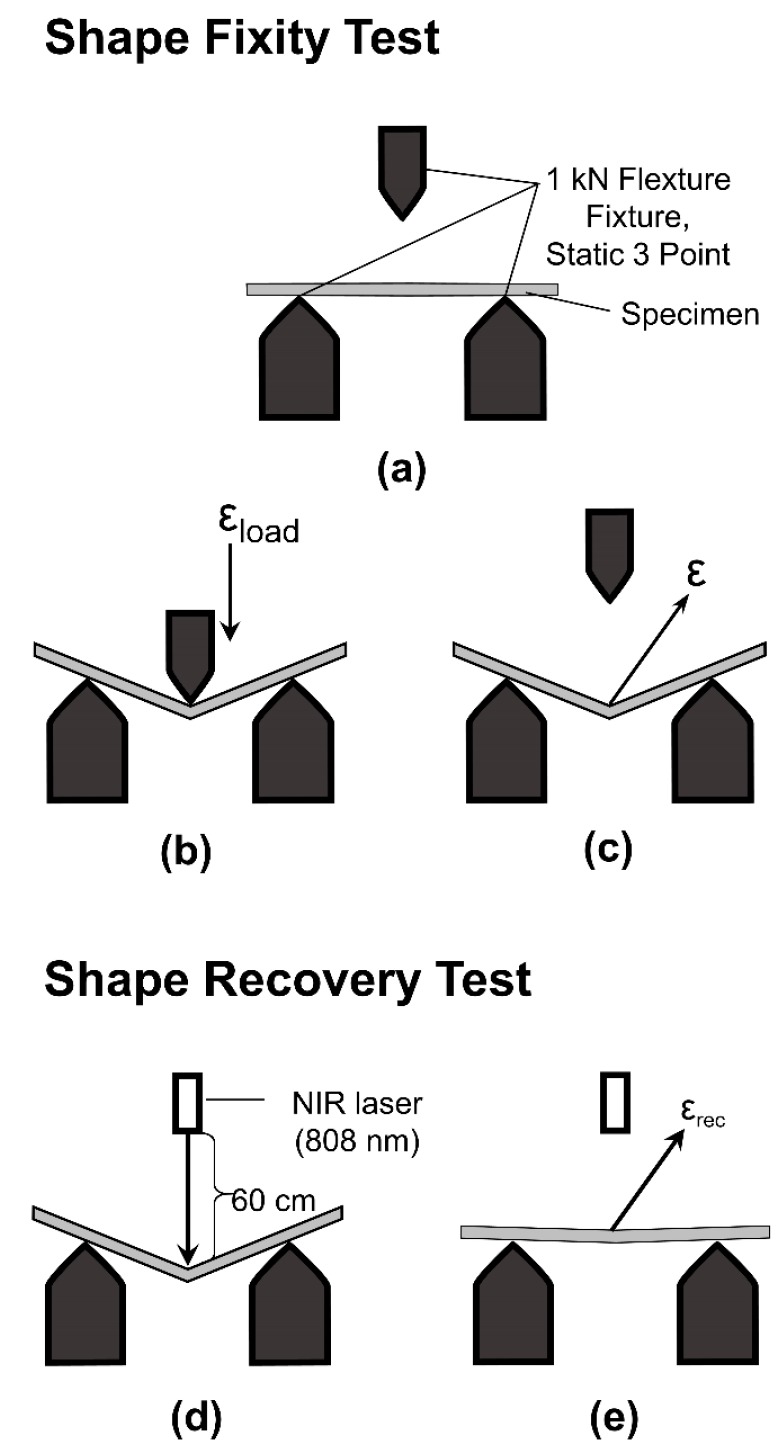
Experimental procedures for the shape fixity test (**a**–**c**) and the shape recovery test (**d**,**e**).

The shape memory properties were tested by a universal testing machine (Model 5567 from Instron Co. Ltd. (Bangkok, Thailand)) with a thermal chamber (Figure 1). The samples with dimensions of 5 mm × 50 mm × 2 mm were tested in a three-point bending mode with a supporting span of 32 mm and a crosshead speed of 1.0 mm/min. Firstly, the temporary shape (Figure 1a) was deformed by applying a bending load of 10% to a sample at *T*_g_ + 20 °C (Figure 1b). The load was continuously applied at *T*_g_ + 20 °C for 20 min. Then, the sample was cooled down to room temperature. The load was then completely removed and a temporary shape was obtained (Figure 1c). The deflection after unloading was then measured and the shape fixity (*R*_f_) of each sample was determined according to Equation (2).

(2) $$R_{f}(\%)=\frac{\varepsilon }{\varepsilon_{\mathrm{load}}}\times 100$$

where *ε*_load_ represents the strain under bending load and *ε* is the fixed strain of the sample upon completion of the deformation step.

In the shape recovery process, the sample was irradiated with a NIR radiation (808 nm) (Hong Kong, China) at the bending edge (Figure 1d). The laser intensity was 500 mW. The distance between the sample and the light source was 60 cm. The fixed shape was subsequently recovered (Figure 1e). The shape recovery (*R*_r_) value was calculated by Equation (3).

(3) $$R_{r}(\%)=\frac{\varepsilon -\varepsilon_{rec}}{\varepsilon }\times 100$$

where *ε*_rec_ is the strain after completion of the recovery step.

## 3. Results and Discussion

**Figure 2:**
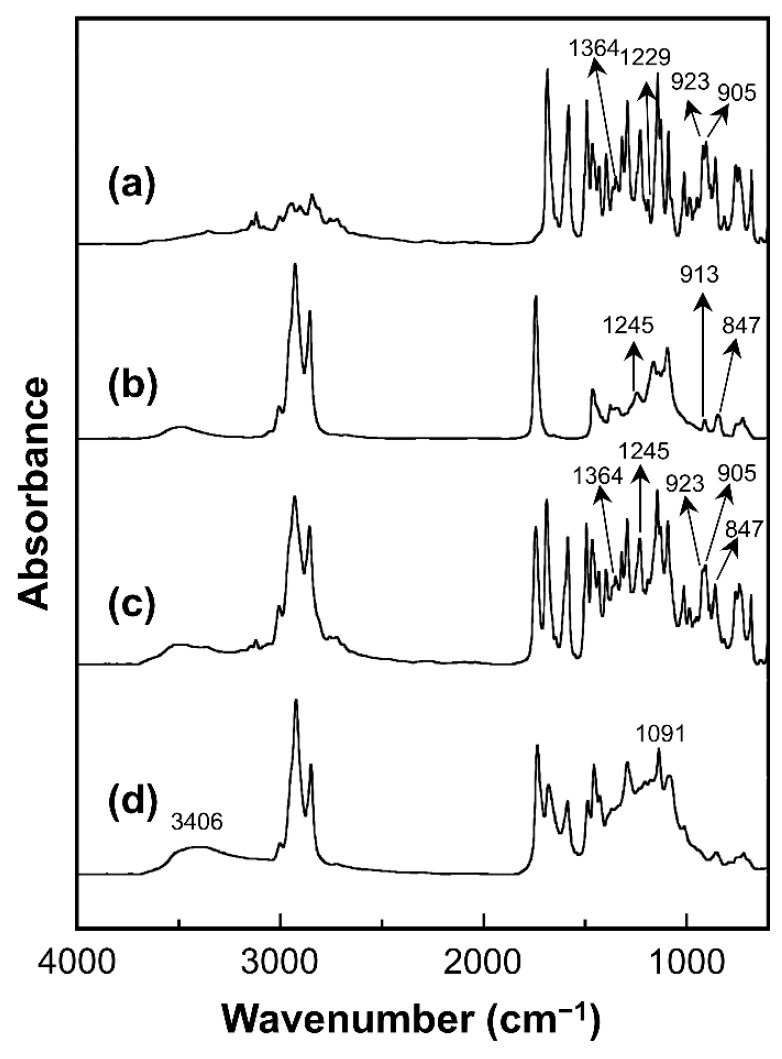
FT-IR spectra of (**a**) V-fa monomer, (**b**) epoxidized castor oil (ECO), (**c**) uncured V-fa/ECO binary mixture, and (**d**) fully cured V-fa/ECO copolymer.

FT-IR spectra of bio-based benzoxazine resin (V-fa), ECO, uncured V-fa/ECO binary mixture, and cured V-fa/ECO copolymer are shown in Figure 2.

**Figure 3:**
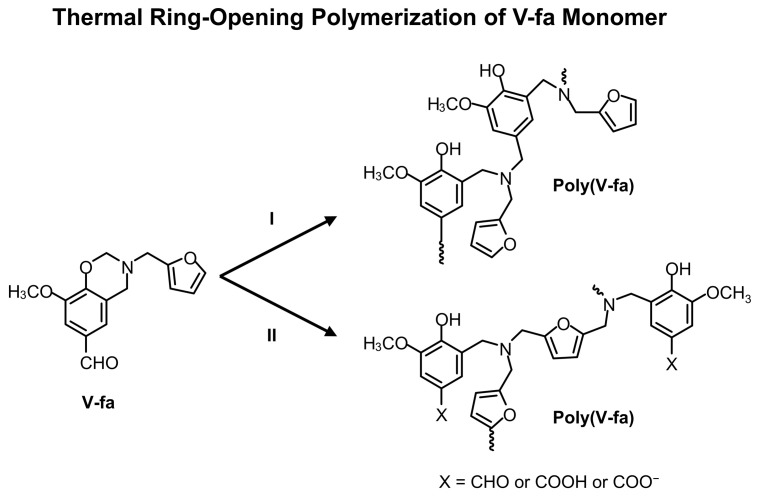
Chemical structures of V-fa monomer and the network structures of poly(V-fa) following ring-opening polymerization.

**Figure 4:**
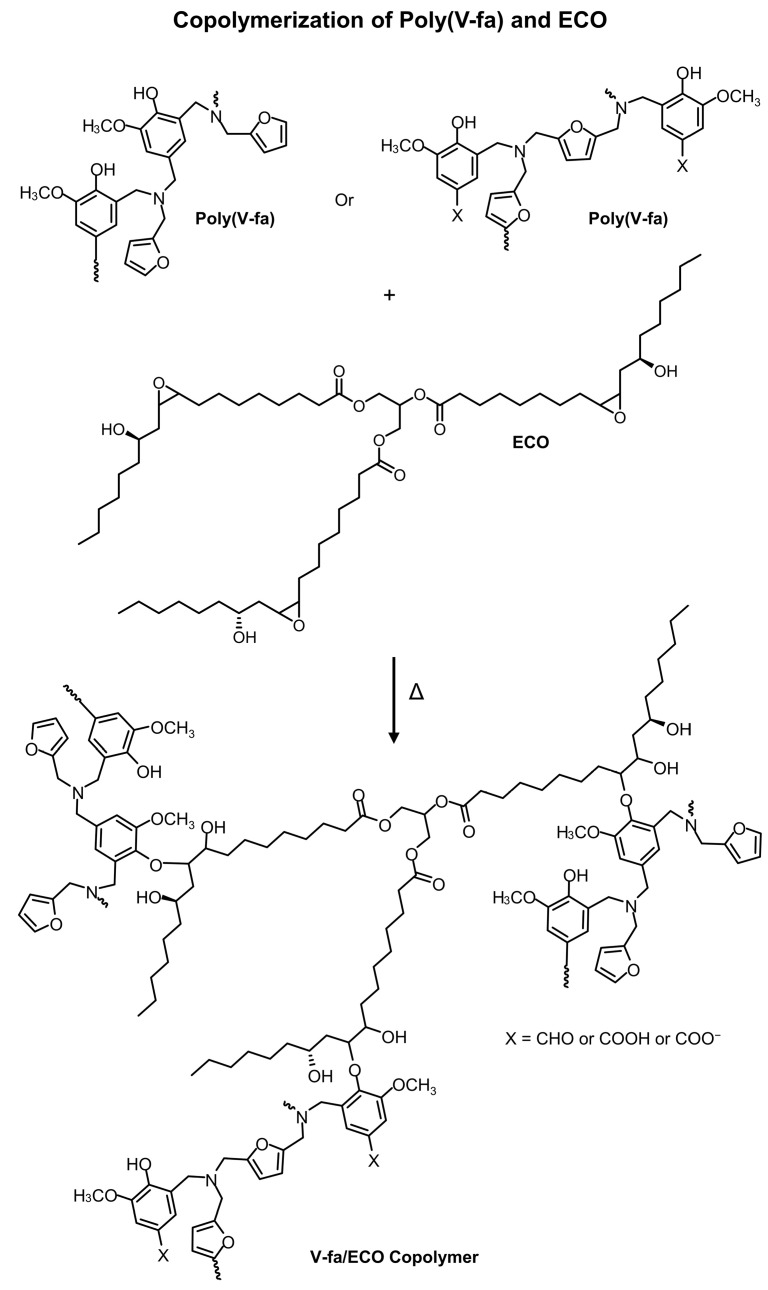
Copolymerization of poly(V-fa) and ECO.

The V-fa monomer exhibited IR absorption peaks at 905, 923, and 1229 cm^−1^ (C–O–C) of an oxazine ring (Figure 2a) [17]. The furan group of V-fa monomer displayed IR absorption peaks spectra at 760, 997, and 1583 cm^−1^ [25]. The IR absorption peak at 1686 cm^−1^ confirmed the presence of carbonyl (C=O) group of vanillin [18]. The spectrum also showed a band at 1364 cm^−1^, attributing to tetra-substituted benzene ring of V-fa [19]. ECO (Figure 2b) exhibited IR absorption peaks of oxirane ring at 1245, 913, and 847 cm^−1^ [14]. The absorption band at 1744 cm^−1^ and 1096 cm^−1^ were assigned to C=O stretching and C–O–C stretching mode of ethers, respectively [14,45]. The IR absorption spectrum of uncured V-fa/ECO mixture (Figure 2c) possessed IR absorption characteristics contributed from both V-fa and ECO. The network formation between V-fa and ECO after thermal curing was also monitored by FT-IR spectroscopy. Figure 2d shows IR absorption spectrum of cured V-fa/ECO copolymer. The polymerization profile employed for thermal curing of V-fa/ECO binary mixture was 150 °C for 1 h, 160 °C for 1 h, 170 °C for 2 h, and 180 °C for 2 h. V-fa underwent thermal ring-opening polymerization and the oxazine ring was opened by the breakage of a C–O bond. V-fa molecules were transformed from a ring structure and interconnected to a three-dimensional network structure as depicted in Figure 3. Electrophilic substitution of carbocation intermediates with another V-fa monomer might occur at the aromatic ring (pathway I) or the furan moiety (pathway II). The absorption bands corresponding to oxazine ring at 905, 923, 1229 cm^−1^ were disappeared, confirming the ring-opening polymerization of V-fa [17]. Poly(V-fa) possessed in situ generated phenolic hydroxyl groups that could be employed to copolymerize with ECO [46]. The copolymerization of poly(V-fa) and ECO is schematically drawn as shown in Figure 4. The copolymerization of ring-opened V-fa and ECO was evidenced by the disappearance of IR absorption peaks of oxirane ring at 1245, 913 and 847 cm^−1^. Epoxide groups can react with the phenolic hydroxyl group of the ring opened V-fa monomers to form new ether linkages and hydroxyl groups in epoxy moieties as clearly seen from the emerging absorption bands at 1091 and 3406 cm^−1^, respectively [14]. Kimura et al. [47] and Rimdusit et al. [48] reports that benzoxazine resin could be employed as a curing agent for epoxy. These results confirmed that V-fa could be synthesized from vanillin, furfurylamine, and paraformaldehyde by the solventless method. In addition, poly(V-fa) could be copolymerized with ECO through the reaction between phenolic hydroxy groups of poly(V-fa) and epoxide groups of ECO.

**Figure 5:**
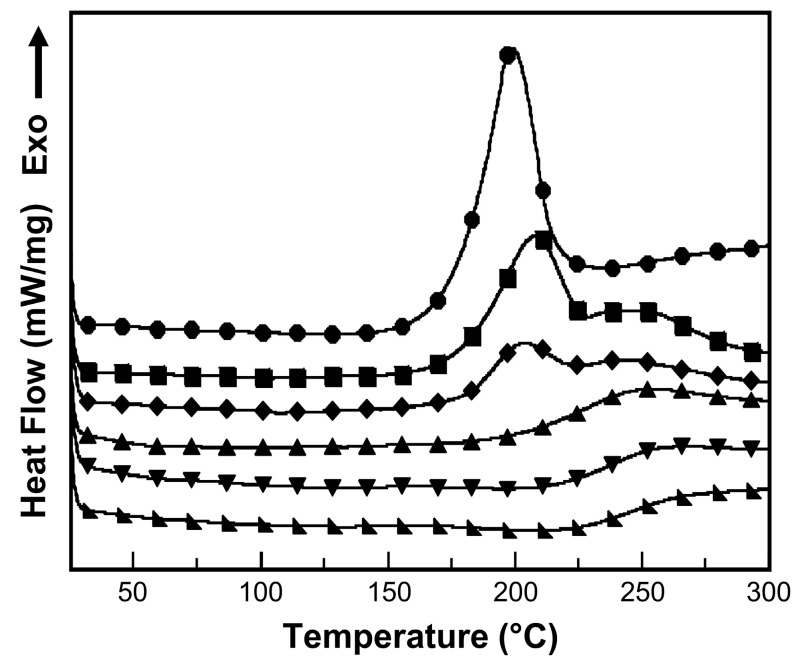
DSC thermograms of (●) pure V-fa and V-fa/ECO binary mixture at 50 wt% of V-fa content after a step thermal curing: (■) uncured binary mixture, (♦) 150 °C/1 h, (▲) 160 °C/1 h, (▼) 170 °C/2 h, and (◣) 180 °C/2 h.

Curing behavior of V-fa monomer and V-fa/ECO mixtures were studied by differential scanning calorimetry (DSC). The DSC thermogram of V-fa monomer is shown in Figure 5. There was a curing exotherm in the range from 181 to 216 °C. The onset and peak exothermic temperatures of V-fa monomer were 181 and 199 °C, respectively. The enthalpy of reaction was 168 J g^−1^. The exothermic peak was assigned to the ring-opening polymerization of V-fa monomer [17]. V-fa monomer exhibited a rather low curing temperature due to the presence of carboxylic groups catalyzing the ring-opening polymerization [14].

Curing behavior of V-fa/ECO copolymer at V-fa mass concentration of 50 wt% at various curing conditions was also investigated by DSC (Figure 5). DSC thermograms of V-fa/ECO copolymer revealed two exothermic peaks, implying that there were two major reactions between V-fa and ECO. The first reaction at the lower temperature was attributed to the thermal ring-opening polymerization reaction of V-fa monomers [17]. The second exothermic peak at a higher temperature was the reaction between phenolic hydroxyl groups of polybenzoxazine (poly(V-fa)) and epoxide groups of the ECO [14]. Curing conversions of the V-fa/ECO copolymer were determined from the partial disappearance of the area under the exothermic peaks in DSC thermograms (Equation (1)). The heat of reactions of the uncured sample was measured to be 160 J/g. After the step curing at 150 °C/1 h, 160 °C/1 h, 170 °C/2 h, and 180 °C/2 h, the heat of reactions decreased to 77.4, 29.9, 17.4, and 8.30 J/g, respectively. The degree of conversions of V-fa/ECO copolymers were 51.7, 81.3, 89.1, and 94.8% after the step curing at 150 °C/1 h, 160 °C/1 h, 170 °C/2 h, and 180 °C/2 h, respectively. The curing conversion of 94.8% was selected for further investigation. Partially bio-based systems of epoxy phenolic novolac (EPN)/cashew nut shell liquid composites reinforced with MWCNTs at curing conversions of 80 to 98% exhibited good shape memory performances as reported by Kasemsiri et al. [49].

**Figure 6:**
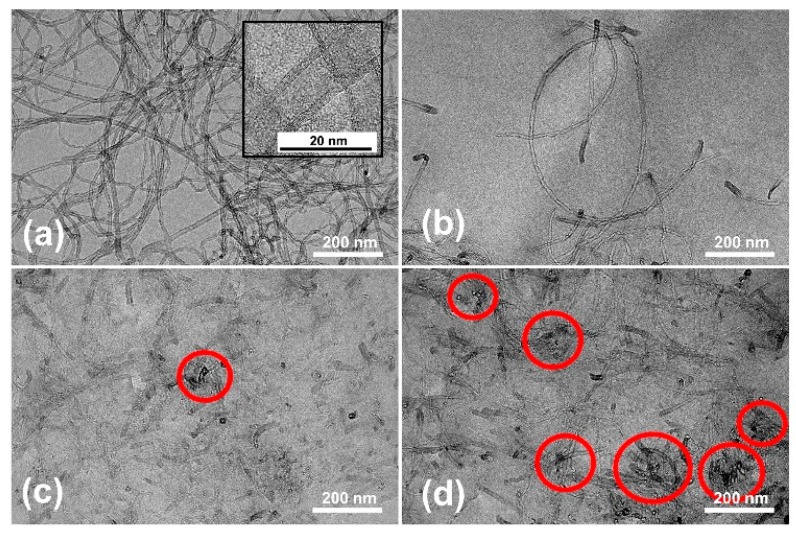
Transmission electron micrographs of (**a**) pure multiwalled carbon nanotubes (MWCNTs) and MWCNT reinforced V-fa/ECO composites at MWCNT contents of (**b**) 0.1 wt%, (**c**) 0.3 wt%, and (**d**) 0.5 wt%.

Morphology of MWCNTs and their distribution within composite specimens were observed by a transmission electron microscope. Transmission electron micrographs of pure MWCNTs and MWCNT reinforced V-fa/ECO composites at 0.1 to 0.5 wt% of MWCNTs are shown in Figure 6.

MWCNTs possessed an average diameter of 10.1 ± 2.3 nm as directly measured from micrographs. Multiwalled characteristics of carbon nanotubes were also confirmed with observed fringe patterns as shown in the inset of Figure 6a [50]. MWCNTs were uniformly dispersed within V-fa/ECO matrix when MWCNT mass concentrations were 0.1 wt% (Figure 6b) and 0.3 wt% (Figure 6c). At high MWCNT mass concentration of 0.5 wt%, some MWCNTs were aggregated within V-fa/ECO matrix (Figure 6d).

**Figure 7:**
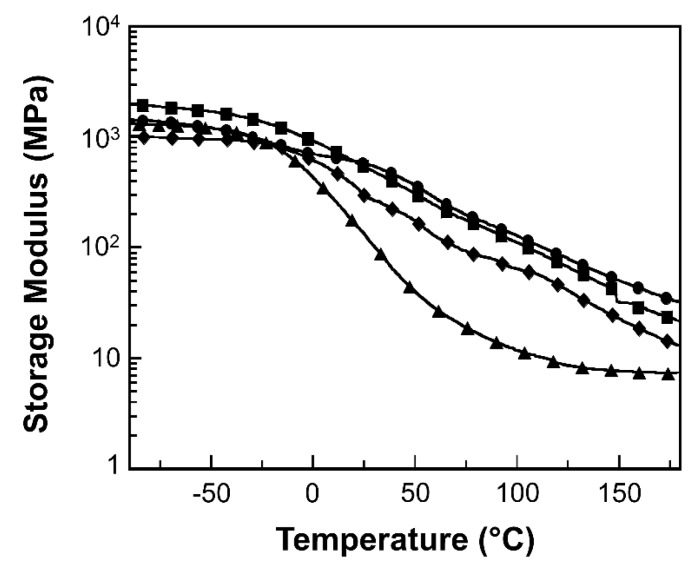
Storage modulus of MWCNT reinforced V-fa/ECO composites at various MWCNT contents of (●) 0 wt%, (▲) 0.1 wt%, (■) 0.3 wt%, and (♦) 0.5 wt%.

Dynamic mechanical properties of MWCNT reinforced V-fa/ECO composites, i.e., storage modulus (E′) and loss tangent (tan δ) were investigated by a dynamic mechanical analyzer (DMA). Figure 7 presents the storage modulus as a function of temperature of MWCNT reinforced V-fa/ECO composites. The glassy state modulus at −100 °C of MWCNT reinforced V-fa/ECO composites at MWCNT contents of 0, 0.1, 0.3, and 0.5 wt% were 1.33, 1.44, 2.01, and 1.03 GPa, respectively. The storage modulus values of MWCNT reinforced V-fa/ECO composites reached the maximum value when MWCNT mass concentration was 0.3 wt% due to the homogeneous dispersion of nanofiller within the polymer matrix [49]. During the V-fa/ECO binary mixture preparation, V-fa monomer might be adsorbed onto the surfaces MWCNTs, facilitating the dispersion of MWCNTs within the mixture [51]. The storage modulus value of the composite at 0.5 wt% drastically reduced due to an aggregation of MWCNTs [52,53]. This phenomena was also observed in MWCNT/polypropylene composites [54]. Kord et al. found that the storage modulus of polyprolypene/reed flour composites decreased when MWCNT contents were 3 to 5 phr because of the agglomeration of MWCNTs [55].

**Figure 8:**
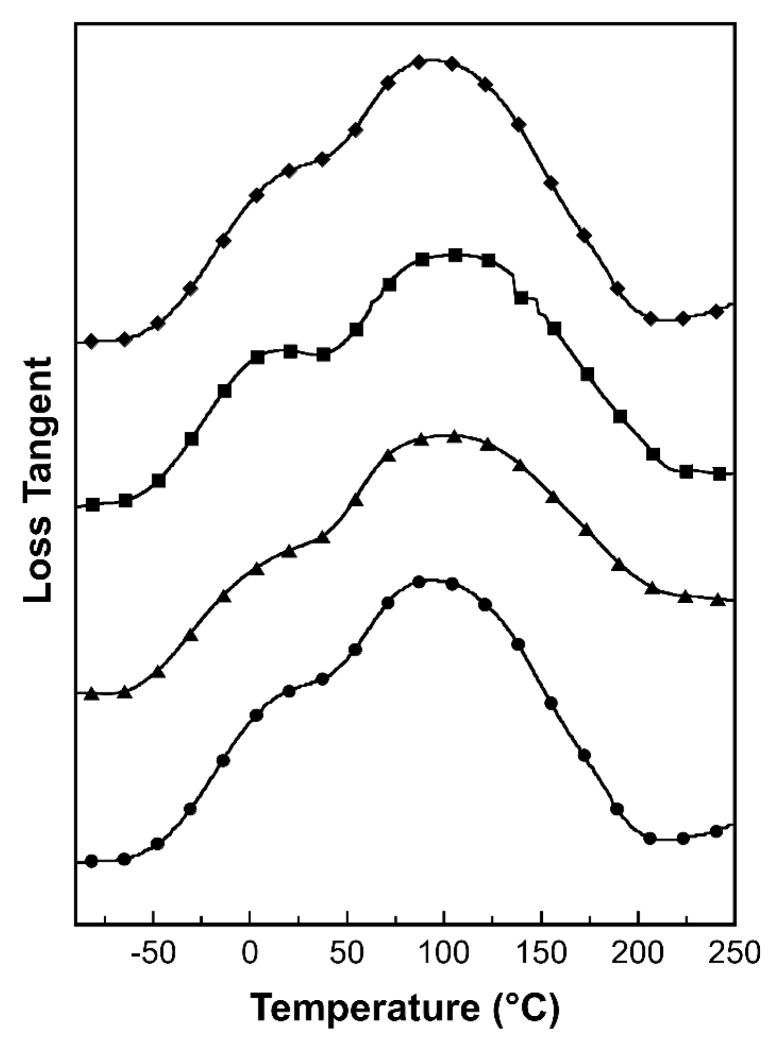
Loss tangent curves of MWCNT reinforced V-fa/ECO composites at MWCNT contents of (●) 0 wt%, (▲) 0.1 wt%, (■) 0.3 wt%, and (♦) 0.5 wt%.

Figure 8 shows loss tangent curves as a function of temperature. *T*_g_ values of the composites were determined from the peak maxima of the tan δ curves. *T*_g_ is an important parameter determining the switching transition temperature of chemically cross-linked amorphous polymer networks. The original shape of polymer specimen could be deformed to the temporary shape with an applying load when the temperature is above *T*_g_. The temporary shape of SMP specimen could be fixed at the temperature below *T*_g_. After exposing to the temperature above *T*_g_, the original shape of SMP specimen could be recovered from the fixed temporary shape [56]. All samples showed tan δ curves possessing two peak maxima, indicating the existence of two-phase structures [57]. The first peak maxima located approximately at 4 °C, attributing to *T*_g_ of the ECO-rich phase of the composites. The other peak maxima in tan δ curves of V-fa/ECO composite reinforced with 0, 0.1, 0.3, and 0.5 wt% of MWCNTs were at 96, 102, 107, and 97 °C, respectively. Fully cured poly(V-fa) exhibited *T*_g_ of 110 °C as determined from the baseline shift of DSC thermogram. Therefore, these peaks should attribute to the V-fa-rich phase. Similar phase behavior was also observed in benzoxazine/DGEBA blending systems [58]. The ECO-rich phase and V-fa-rich phase played a role as the soft segment or the reversible switching phase and the permanent netpoints of MWCNT reinforced V-fa/ECO composites, respectively [14]. The highest *T*_g_ was observed for V-fa/ECO composites reinforced with 0.3 wt% MWCNT due to the uniform dispersion of reinforcing fillers within the copolymer matrix. An optimal addition of MWCNTs to V-fa/ECO copolymer could limit the molecular chain mobility and enhance the *T*_g_ [59]. The small drop in *T*_g_ of the composite with 0.5 wt% of MWCNT was due to the agglomeration of MWCNTs.

**Figure 9:**
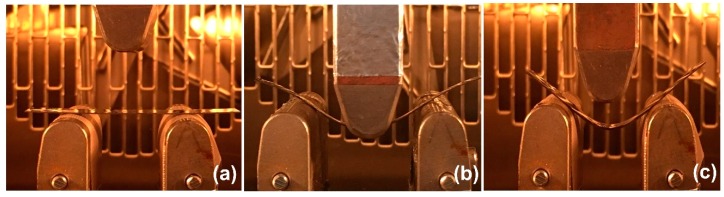
Photographs showing shape fixity process under a three-point bending load for MWCNT reinforced V-fa/ECO composites: (**a**) the original rectangular shape, (**b**) deformed temporary shape at *T*_g_ + 20 °C with 10% bending, and (**c**) fixed temporary shape at room temperature.

Shape memory performances of MWCNT reinforced V-fa/ECO composites were evaluated utilizing three principal parameters, i.e., shape fixity, shape recovery, and shape recovery time. Shape fixity (*R*_f_) indicates the ability of SMPs to memorize the fixed or temporary shape. The shape fixity of specimens was examined by a universal testing machine with a three-point bending mode (flexural mode). The original rectangular shape of MWCNT reinforced V-fa/ECO composites (Figure 9a) could be deformed under a three-point bending load (10% bending) at *T*_g_ + 20 °C (Figure 9b). The temporary shape of MWCNT reinforced V-fa/ECO composites could be fixed when the temperature of the specimen was at room temperature (Figure 9c).

**Figure 10:**
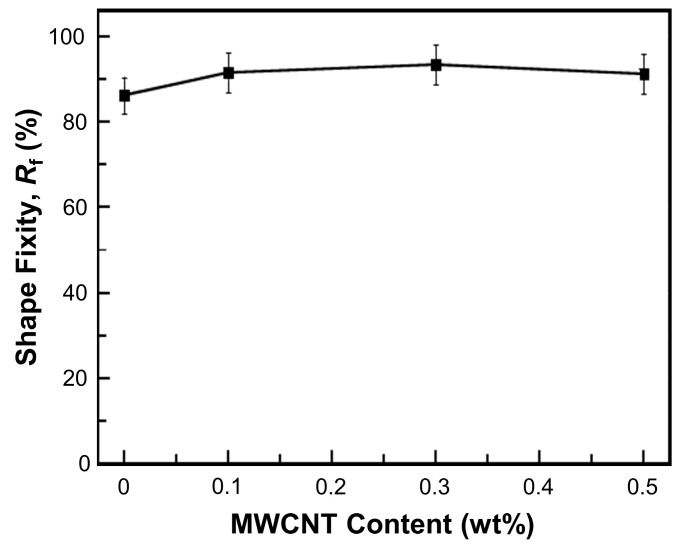
Shape fixity of MWCNT reinforced V-fa/ECO composites at various MWCNT contents.

Shape fixity values under a three-point bending load of MWCNT reinforced V-fa/ECO composites are shown in Figure 10. The shape fixity values of MWCNT reinforced V-fa/ECO composites at 0, 0.1, 0.3, and 0.5 wt% MWCNTs were 86, 92, 93, and 91, respectively. Shape fixity values were significantly enhanced with increasing MWCNT contents up to 0.5 wt%. MWCNTs possibly possessed good interaction with V-fa/ECO matrix and functioned as an additional fixed phase, improving the shape fixity of the composites [51,60]. Similar behavior was also observed in MWCNT-filled shape memory polyurethane nanocomposites [61]. The shape fixity value of MWCNT reinforced V-fa/ECO composites at 0.5 wt% MWCNTs was dropped to 91% because the excess MWCNT mass concentration could induce aggregation of the MWCNTs [62].

**Figure 11:**
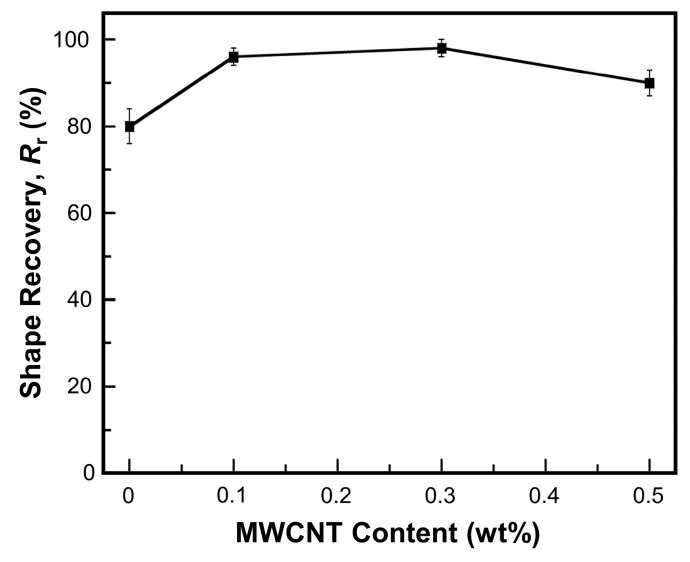
Shape recovery under NIR-actuation of MWCNT reinforced V-fa/ECO composites at various MWCNT contents.

Shape recovery (*R*_r_) is the parameter that used to reflect how well an original shape of SMP specimen is memorized. Shape recovery under NIR-actuation of MWCNT reinforced V-fa/ECO composites at various weight percentages are shown in Figure 11. Shape recovery of MWCNT reinforced V-fa/ECO composites at MWCNT weight percentages of 0, 0.1, 0.3, and 0.5 were 80, 96, 98, and 92%, respectively. MWCNTs efficiently enhanced shape recovery of V-fa/ECO composites when compared to the unfilled specimen because MWCNTs could effectively absorb light and transform into heat distributed within the polymer matrix [16,63]. Addition of 0.3 wt% of MWCNTs maximized shape recovery of the composites to 98%. MWCNTs might possess good interaction with permanent netpoints consisting of V-fa-rich phase, enhancing the storage of internal stress during shape deformation and providing high recovery force during the shape recovery process [64]. However, the shape recovery value of MWCNT reinforced V-fa/ECO composite was dropped to 92% when the MWCNT weight percentage was 0.5 wt% due to the aggregation of the MWCNTs. Similar behavior was also observed in MWCNT-filled shape memory epoxy phenolic novolac and cashew nut shell liquid [49] and polyurethane/MWCNT composites [64].

**Figure 12:**
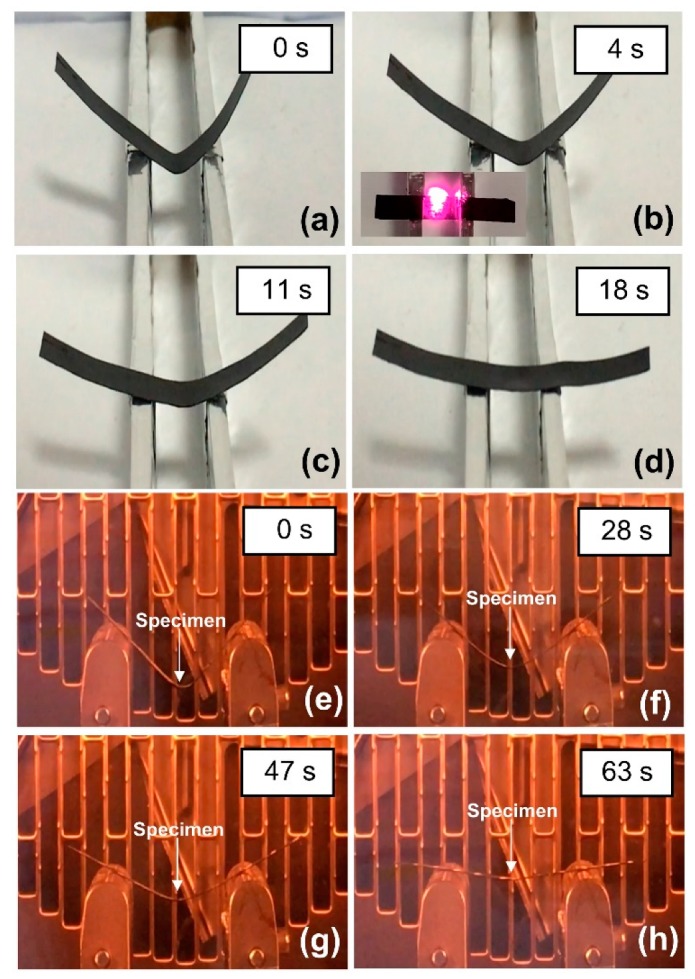
Snapshots of shape recovery process of MWCNT reinforced V-fa/ECO composite at 0.3 wt% of MWCNTs under (**a**–**d**) NIR-actuation and (**e**–**h**) thermal heating.

**Figure 13:**
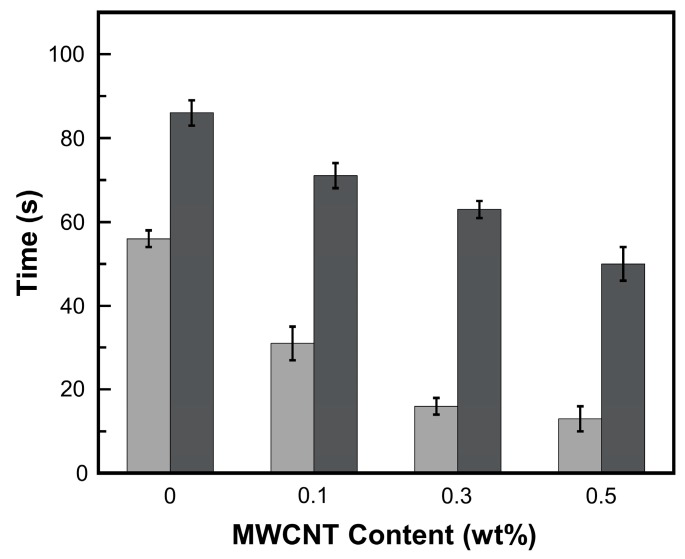
**Shape** recovery time under (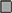) NIR laser triggering and (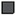) thermal heating of MWCNT reinforced V-fa/ECO composites at 0, 0.1, 0.3, and 0.5 wt% of MWCNTs.

Snapshots of shape recovery process under NIR-actuation and thermal heating of 0.3 wt% MWCNT reinforced V-fa/ECO composites are shown in Figure 12. The temporary shape was formed by bending the original rectangular shape of shape memory composite at 90°. The shape recovery process was recorded with a digital camera. The temporary shape of MWCNT reinforced V-fa/ECO composite could be recovered to the original shape under NIR actuation (Figure 12a–d) and thermal heating (Figure 12e–h). With NIR laser triggering, shape recovery time of MWCNT reinforced V-fa/ECO composites at MWCNT contents of 0, 0.1, 0.3, and 0.5 wt% were 51, 36, 16, and 13 s, respectively (Figure 13). These results demonstrated that NIR laser actuation could be employed for triggering shape memory effects of MWCNT reinforced V-fa/ECO composites fabricated based on renewable resources. MWCNTs played a role as efficient NIR light absorber, transforming absorbed light into heat and distributed to the polymer matrix [16,63]. MWCNTs at 0.3 wt% could raise temperature of MWCNT-poly (lactic acid) up to 100 °C within 16 s under NIR lamp illumination of 275 W [65]. In our system, MWCNTs could induce the thermal heating up to 120 °C within the V-fa/ECO matrix at 0.3 wt% MWCNT as measured with a thermocouple. The temperature induced by photothermal heating with NIR laser exceeded the transition temperature of 0.3 wt% MWCNT reinforced V-fa/ECO composites (107 °C), triggering the shape recovery process. In addition, shape recovery time of MWCNT reinforced V-fa/ECO composites decreased with increasing MWCNT mass concentrations. The addition of MWCNTs could accelerate the shape recovery process due to the enhanced thermal conductivity of MWCNT reinforced composites [66]. Yu et al. [67] also reported that Veriflex-S^®^ VF62/CNTs composites exhibited shorter recovery time when CNTs mass concentration increased due to the minimized thermal conduction pathway within the composites. Even though the shape recovery process of MWCNT reinforced V-fa/ECO composites could be triggered under thermal heating, the shape recovery time was significantly longer. The shape recovery time of MWCNT reinforced V-fa/ECO composites under thermal heating were 86, 71, 63, and 50 s when MWCNT mass concentrations were 0, 0.1, 0.3, and 0.5 wt%, respectively (Figure 13).

**Figure 14:**
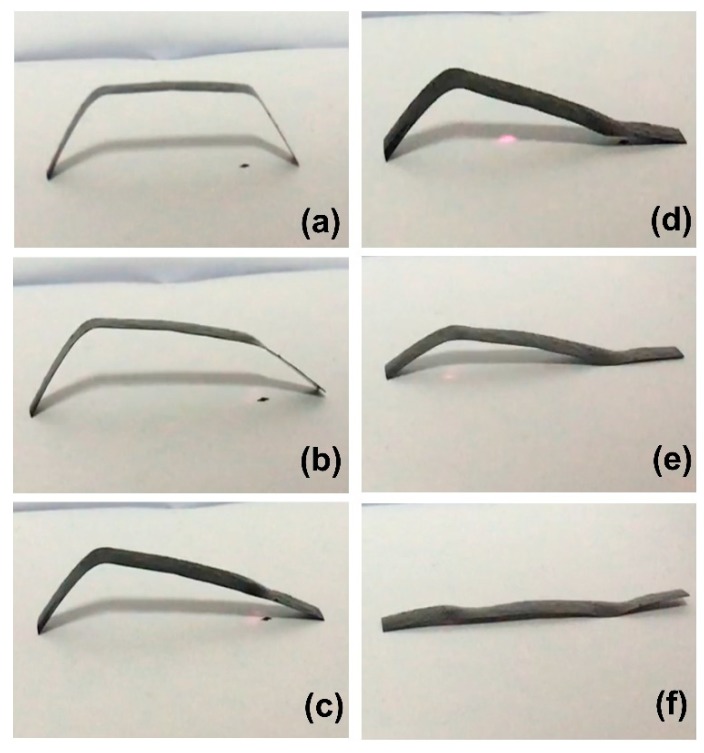
Snapshots of site-selective shape recovery process of MWCNT reinforced V-fa/ECO composite at 0.3 wt% of MWCNTs: (**a**) the deformed “U” shape of the composite, (**b**,**c**) initial irradiation of the deformed composite with NIR laser at the first crease, (**d**,**e**) subsequent irradiation of the partially recovered composite with NIR laser at the other crease, and (**f**) the fully recovered original shape of the composite.

MWCNT reinforced V-fa/ECO composite at 0.3 wt% of MWCNTs also exhibited site-selective shape recovery process under NIR actuation (Figure 14). The original shape of the composite was deformed into “U” shape with two separated creases (Figure 14a). NIR laser could be employed for stepwise recovery of the composites simply by irradiating at the specified point (Figure 14b–f) without disturbing the other crease. In addition, the shape recovery process could be remotely initiated and terminated by turning on or turning off the NIR laser. These operations could not be performed with conventional thermal activation.

## 4. Conclusions

Multiwalled carbon nanotube reinforced vanillin-furfurylamine-containing benzoxazine (V-fa)/epoxidized castor oil (ECO) composites with NIR laser actuated shape memory effects were successfully prepared. V-fa monomer was successfully synthesized from vanillin, furfurylamine, and paraformaldehyde using the solventless approach as confirmed by FT-IR spectroscopy. The curing behavior as determined from differential scanning calorimetry of MWCNT reinforced V-fa/ECO composites exhibited two exothermic peaks corresponding to the thermal ring-opening polymerization reaction of V-fa monomers and copolymerization of in situ generated phenolic hydroxyl groups of polybenzoxazine (poly(V-fa)) and epoxide groups of ECO. MWCNT reinforced V-fa/ECO composites exhibited shape memory effects and NIR laser actuation could be employed to stimulate the shape recovery process of the composites. An addition of MWCNTs from 0.1 to 0.3 wt% significantly improved thermal, dynamic mechanical, and shape memory properties of MWCNT reinforced V-fa/ECO composites. The composites exhibited shape fixity from 92 to 93%, shape recovery from 96 to 98%, and shape recovery time from 36 to 16 s after an addition of MWCNTs from 0.1 to 0.3 wt%. The temporary shape of MWCNT reinforced V-fa/ECO composite was efficiently recovered to the original shape under NIR actuation when compared to the shape recovery process under thermal triggering. Reinforcing MWCNTs could effectively convert the absorbed NIR light into thermal energy and distribute the energy within the polymer matrix. Furthermore, remote activation and site-specific shape recovery process under NIR irradiation could be realized for MWCNT reinforced V-fa/ECO shape memory composites.
